# DeepIndel: An Interpretable Deep Learning Approach for Predicting CRISPR/Cas9-Mediated Editing Outcomes

**DOI:** 10.3390/ijms252010928

**Published:** 2024-10-11

**Authors:** Guishan Zhang, Huanzeng Xie, Xianhua Dai

**Affiliations:** 1 College of Engineering, Shantou University, Shantou 515063, China; gszhang@stu.edu.cn (G.Z.); 22hzxie@stu.edu.cn (H.X.); 2 School of Cyber Science and Technology, Sun Yat-sen University, Shenzhen 518107, China

**Keywords:** CRISPR/Cas9, BERT, repair outcomes, Deep SHAP

## Abstract

CRISPR/Cas9 has been applied to edit the genome of various organisms, but our understanding of editing outcomes at specific sites after Cas9-mediated DNA cleavage is still limited. Several deep learning-based methods have been proposed for repair outcome prediction; however, there is still room for improvement in terms of performance regarding frameshifts and model interpretability. Here, we present DeepIndel, an end-to-end multi-label regression model for predicting repair outcomes based on the BERT-base module. We demonstrate that our model outperforms existing methods in terms of accuracy and generalizability across various metrics. Furthermore, we utilized Deep SHAP to visualize the importance of nucleotides at various positions for DNA sequence and found that mononucleotides and trinucleotides in DNA sequences surrounding the cut site play a significant role in repair outcome prediction.

## 1. Introduction

CRISPR/Cas9 (clustered regularly interspaced short palindromic repeats-associated protein 9) is a remarkable genome editing technique with promising potential for genetic manipulation applications [1]. This system consists of a single guide RNA (sgRNA) and a Cas9 endonuclease [2]. The sgRNA guides Cas9 to genomic loci, which direct it to a 20 bp spacer sequence immediately upstream of the protospacer adjacent motif (PAM) [3]. Consequently, double-strand DNA breaks (DSBs) are generated at target loci [4]. Homology-directed repair (HDR) and classical nonhomologous-mediated end joining (NHEJ) are two major pathways for DSB repair [5]. HDR uses homologous template sequences to repair the DSB, potentially introducing programmed edits by the repair template. In contrast, NHEJ directly rejoins the broken ends, often perfectly but sometimes introducing errors that typically lead to the formation of short insertions or deletions (indels) [6]. Additionally, evidence suggests that microhomology-mediated end joining (MMEJ) can result in larger deletions at regions of microhomology [7] and complex events [8]. DSBs are harmful to cells because if left unrepaired, they can further lead to chromosomal abnormality. Several studies have provided insights into the mechanisms affecting Cas9-induced DNA editing [9,10,11]. However, our understanding of how Cas9 interacts with sgRNAs and how cells respond to Cas9-mediated DNA damage is limited, making the editing outcome unpredictable and often preventing a rational use of this technique. Thus, increasing our knowledge of the mechanisms regulating these interactions is critical to maximize the potential and safety of CRISPR-based applications.

Several studies have shown that the repair outcomes of Cas-induced DSBs are predictable and nonrandom [12,13,14,15,16]. The protospacer sequence plays a significant role in determining DSB repair outcomes [9,17]. For example, Overbeek et al. [9] targeted seven sites in the genome that can be uniquely amplified by a spacer sequence and observed that the repair outcomes for these sites were very similar. Given the nonrandom nature of Cas9-mediated DSB repair, abundant public data can be used to develop machine learning algorithms to predict the repair outcomes [9,15,18,19]. For instance, Shen et al. [15] introduced Delphi, which combines k-nearest neighbors with two neural networks to predict the genotypes and frequencies of editing products from target DNA sequences with high accuracy in five human and mouse cell lines. Allen et al. [14] proposed a multi-class logistic regression model named FORECasT to predict in-frame mutations from a given sgRNA with high accuracy. Chakrabarti et al. [13] proposed an artificial neural network to predict CRISPR-mediated genome editing outcome using sgRNA sequences alone. The authors demonstrated that precise targets and editing outcomes are predictable based on simple rules that mainly depend on the fourth nucleotide upstream of the PAM. Chen et al. [20] developed a logistic regression-based Lindel using only the local sequence context to predict the distribution of mutational outcomes. Ryan et al. [16] created a gradient boosting decision tree model named SPROUT to predict the repair outcomes in primary T cells. The proposed model takes the protospacer sequence plus PAM as an input and predicts the length, probability, and sequence of nucleotide insertions and deletions. However, the accuracy of machine learning-based methods varies widely depending on the constructed features. Additionally, the hand-crafted features may result in redundancy, thereby contributing to poor results.

Deep learning can automatically learn high-level feature representations from input data, thus avoiding manual feature engineering. A Convolutional Neural Network (CNN) [21] can capture local correlations in sequences and learn abstract biological sequence features. For example, Li et al. [22] proposed a deep multi-task CNN-based framework named CROTON to predict Cas9-induced editing outcomes. The authors applied in silico saturated mutagenesis analysis to reveal local sequence determinants for the editing outcomes and found that nucleotides surrounding the PAM site are strong determinants of the editing outcomes. Recurrent neural networks (RNNs) [23] such as Long and Short-Term Memory (LSTM) networks [23] and Gated Recurrent Units (GRUs) [24] can capture the contextual information in biological sequences with varying lengths. For instance, Liu et al. [25] proposed Apindel, a bidirectional LSTM (BiLSTM)-based framework incorporating an attention mechanism [26] for editing outcome prediction. Their results illustrated that applying the attention mechanism can focus on key information in the sequence to improve model performance.

Recently, Bidirectional Encoder Representations from Transformers (BERT) [27] has made great progress in the domain of natural language processing (NLP). It is based on a multilayer bidirectional transformer encoder to generate accurate language representations by integrating multiple transformer blocks. Previous reports have shown that BERT achieves satisfactory performance for sequence data analysis. For instance, Ji et al. [28] introduced a BERT-based DNABERT for predicting promoters, splice sites, and transcription factor binding sites. Luo et al. [29] proposed a deep learning framework, which combines CNNs, bidirectional GRUs (BiGRUs), and BERT for off-target prediction. The authors demonstrated that incorporating BERT allows for high performance. In addition, previous studies showed that sequences adjacent to the cut site are an important determinant of Cas9 repair outcomes [12,13,14,18]. The success of previous reports motivated us to apply BERT for repair outcome prediction. Moreover, although existing deep learning-based methods (e.g., CROTON and Apindel) achieve good performance on insertions and deletions, there is still room for improving the performance regarding frameshift frequency and interpretability.

Here, we present DeepIndel, an end-to-end multi-label regression model designed for predicting Cas9-induced repair outcomes. The BERT module in DeepIndel is used for the feature extraction of the input numerical sequence of size 179, obtained by encoding a sequence of length 60 bp. Subsequently, the output of the dense layer is flattened and then fed into two fully connected layers. The final output layer consists of six neurons representing the prediction of each repair label, including deletion frequency, 1 bp insertion frequency, 1 bp deletion frequency, 1 bp frameshift frequency, 2 bp frameshift frequency, and total frameshift frequency. This paper presents the following four contributions. First, we proposed a novel token dictionary for presenting each target DNA sequence as a numerical sequence. Second, we developed a deep learning framework named DeepIndel, which is based on the BERT-based module, to predict repair outcomes. Third, we applied visualization analysis methods using Deep SHAP to reveal which nucleotide positions of the target DNA sequence help our model to make predictions. We observed that mononucleotides and trinucleotides surrounding the cut site play a significant role in our model to predict repair outcomes. This visualization is particularly useful for sgRNA optimization in practical applications. Experiments on public datasets showed that our model consistently outperforms existing methods in terms of accuracy, generalizability, and interpretability.

## 2. Results

### 2.1. Ablation Study Shows the Importance of the Token Dictionary and BERT Module

We first evaluated the effectiveness of the proposed token dictionary and BERT module in DeepIndel, made for predicting repair outcomes. To this end, we conducted a comparative analysis of three variants derived from our model by modifying its components as follows (Table 1). Specifically, we first examined the importance of the BERT module in our model. To this end, we used CNNs and BiLSTM to replace the BERT module in our DeepIndel architecture and obtained DeepIndel-CNN and DeepIndel-LSTM, respectively. Second, we analyzed the importance of the proposed token dictionary by replacing the BERT module in DeepIndel, with BERT weights obtained using semantic dictionary and semantic text pre-training (DeepIndel-pre-trained).

**Figure 1:**
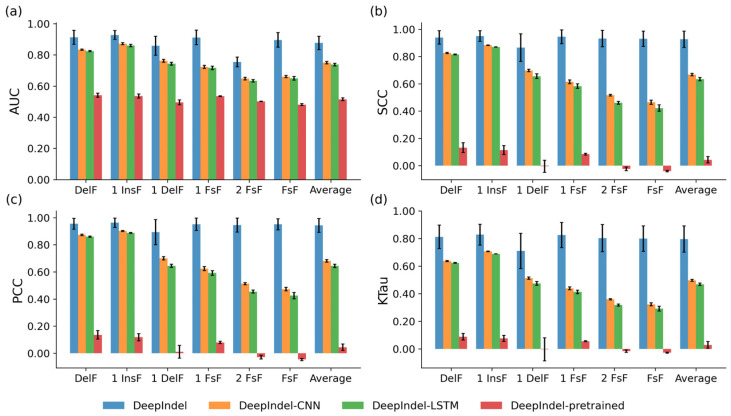
Performance comparison of DeepIndel with three model variants on the K562 dataset under 5-fold cross-validation. The performance comparison in terms of (**a**) AUC, (**b**) SCC, (**c**) PCC, and (**d**) KTau. The prediction methods are arranged vertically, whereas the repair outcomes are arranged horizontally. DelF, deletion frequency; 1 InsF, 1 bp insertion frequency; 1 DelF, 1 bp deletion frequency; 1 FsF, 1 bp frameshift frequency; 2 FsF, 2 bp frameshift frequency; FsF, total frameshift frequency, and the average of all prediction tasks. These representations also apply to Figures 2–5 and Supplementary Figures S1–S5. Error bars represent the standard deviation.

We conducted the ablation experiments on the K562 dataset (N = 35129) under five-fold cross-validation. Each entry in the dataset consisted of a 60 bp target sequence and six corresponding labels, including deletion frequency (DelF), 1 bp insertion frequency (1 InsF), 1 bp deletion frequency (1 DelF), 1 bp frameshift frequency (1 FsF), 2 bp frameshift frequency (2 FsF), and total frameshift frequency (FsF). We randomly divided the dataset into a training set and a testing set, with the proportion of 90% and 10%. In addition, we used the receiver operating characteristic curve (AUC), Spearman correlation coefficient (SCC), Pearson correlation coefficient (PCC), and Kendall’s rank correlation coefficient (KTau) to evaluate the performance of the models. Figure 1 shows the quantitative results of the comparison between DeepIndel and three model variants, and each result in the figure was obtained by aggregating the entire test set, including DelF, 1 InsF, 1 DelF, 1 FsF, 2 FsF, and FsF, and the average of all the labels. Overall, DeepIndel demonstrates superior performance over the other three variants, achieving an averaged AUC of 0.877, SCC of 0.927, PCC of 0.943, and KTau of 0.796 for all prediction tasks. This demonstrates improvements of 12.7%, 26%, 26.2%, and 29.9% in terms of the four quantitative indicators in comparison to DeepIndel-CNN. In addition, we observed that replacing the BERT with BiLSTM in our model (DeepIndel-LSTM) results in declines of 13.9%, 29.2%, 29.9%, and 32.7% in these four indicators, respectively. Furthermore, applying the pre-trained BERT-base model and fine-tuning it (DeepIndel-pre-trained) on the K562 dataset resulted in averaged AUC, SCC, PCC, and KTau values of 0.515, 0.043, 0.045, and 0.029, with reductions of 36.2%, 88.4%, 89.8%, and 76.7%, respectively, compared with DeepIndel training from scratch. This is expected given that the pre-trained BERT-base model is trained on a semantic dictionary, which differs from the sequence data. Collectively, these results demonstrate a clear advantage of applying the proposed token dictionary and the BERT-based module in our model and training it from scratch for extracting abstract features of the DNA sequences, further contributing to the prediction power.

### 2.2. DeepIndel Can Accurately Predict Cas9-Mediated Repair Outcomes

**Figure 2:**
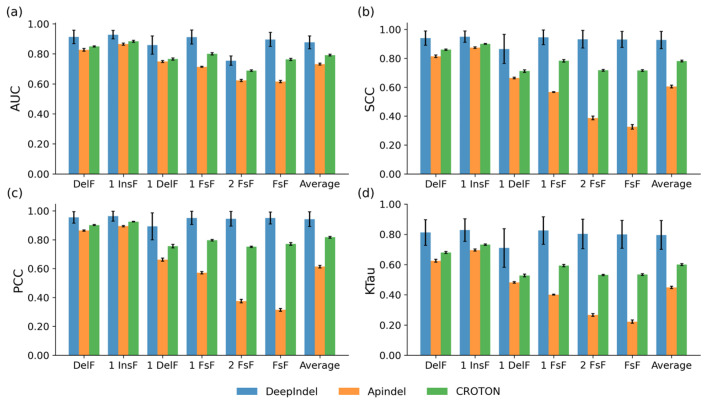
The bar graphs show averaged (**a**) AUC, (**b**) SCC, (**c**) PCC, and (**d**) KTau values of DeepIndel and two deep learning-based methods on the K562 dataset under 5-fold cross-validation. The Error bars represent the standard deviation.

Next, we demonstrate that DeepIndel can more accurately predict the repair outcomes compared with existing methods through experiments on public datasets. To this end, we compared it with two deep learning-based methods (e.g., Apindel [25] and CROTON [22]) on three datasets, including K562, HEK293t (N = 4591), and a T cell dataset (N = 1603). For each dataset, the training set and testing set were generated in the same way as described in Section 2.1. The mean individual performances on K562 dataset are summarized in Figure 2. We observed that DeepIndel performs best on this dataset for all prediction tasks in terms of four performance metrics, achieving a mean AUC of 0.877, SCC of 0.927, PCC of 0.943, and KTau of 0.796, with 8.4%, 14.6%, 12.6%, and 19.6% increases, respectively, compared to the second-best method, CROTON. We also found that DeepIndel outperforms other methods with wide margins on three frameshift frequency prediction tasks, including 1 FsF, 2 FsF, and FsF. Supplementary Figure S1 shows the correlations between true frequencies and predicted frequencies in terms of all prediction tasks on the K562 test set of Deepindel and two deep learning-based methods. For the sake of clarity, DeepIndel outperformed others on all prediction tasks, especially on frameshift frequency tasks. Similar results were also observed on the HEK293t and T cell datasets (Supplementary Figures S2 and S3). Together, these observations indicate that DeepIndel consistently outperforms competing methods for predicting Cas9-mediated repair outcomes, especially in frameshift frequency prediction.

### 2.3. Assessment of Generalizability Performance under Cross-Dataset Test

**Figure 3:**
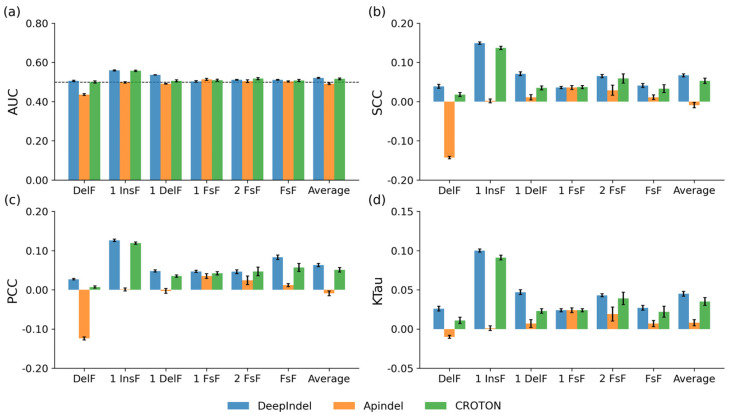
The bar graphs show the (**a**) AUC, (**b**) SCC, (**c**) PCC, and (**d**) KTau of DeepIndel with two existing deep learning-based methods (e.g., Apindel and CROTON) under cross-dataset validation tests. The models were trained on the K562 dataset and their performance was tested on the T cell dataset. The error bars represent the standard deviation. The dotted line in (**a**) indicates an AUC of 0.5.

In this section, we aim to evaluate the generalizability of our model. We compared it to two methods, Apindel and CROTON, using a cross-dataset validation test, which can ensure that the models were tested on unseen data and avoided overfitting. This procedure was also adapted in a previous study [25]. For each method, we used the K562 and HEK293t datasets as training sets to train the models. The T cell dataset was applied as an independent test set. Figure 3 depicts the resulting AUC, SCC, PCC, and KTau for each model trained on the K562 dataset and tested on the T cell dataset. Overall, DeepIndel performed best in terms of all performance metrics except for the 1 FSF prediction task. The second-best CROTON showed comparable performance to DeepIndel in terms of AUC and SCC at 1 FSF prediction. In comparison to CROTON, DeepIndel showed 0.005, 0.014, 0.012, and 0.010 increases in the AUC, SCC, PCC, and KTau for the average of all prediction tasks, respectively. A similar observation was also made when models were trained on the HEK293t dataset and tested on the T cell dataset (Supplementary Figure S4). Collectively, these results illustrate that DeepIndel outperforms the competing methods, thereby showing its superior generalizability.

### 2.4. Nucleotide Contributions Revealed by Deep SHAP

**Figure 4:**
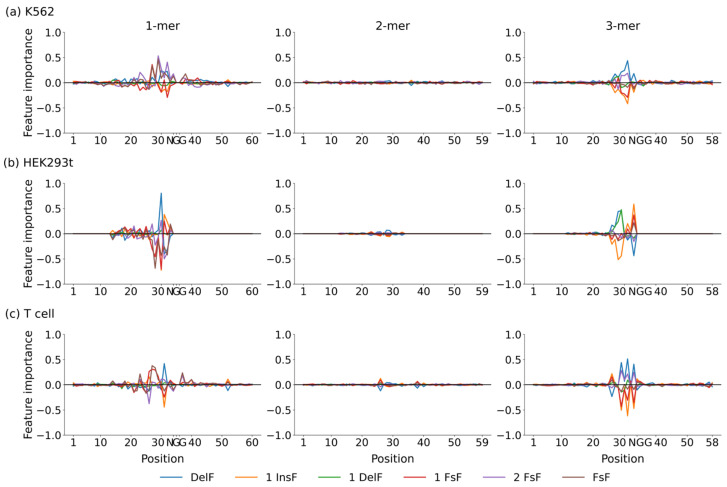
Visualization of the feature importance of 1-mer, 2-mer, and 3-mer sequences at each position learned by DeepIndel on the (**a**) K562, (**b**) HEK293t, and (**c**) T cell datasets, respectively. A positive value indicates a favored feature, whereas a negative value means a disfavored feature.

To understand what our network has learned, we applied Deep SHAP to estimate the position-dependent nucleotide contributions to DeepIndel. The contributions of each position-dependent sequence, embedded using various *k*-mers (*k* = 1, 2, 3), to the repair outcomes were computed from the mean value of that position across all the training target DNA sequences. To make the nucleotides comparable across the K562, HEK293t, and T cell datasets, Deep SHAP values were rescaled by standardization. Figure 4 depicts the positional effects of features extracted from target DNA sequence embedded using 1-mer, 2-mer, and 3-mer on repair outcomes learned by our model. As expected, we observed that the nucleotides (1-mer) around position 30 show higher feature importance values. The same trends were observed when being learned by trinucleotides (3-mer). These position-dependent patterns align with previous findings that positions at and near the cut site (the fourth nucleotide upstream the PAM) are the most important [15,18,22] for repair outcomes.

**Figure 5:**
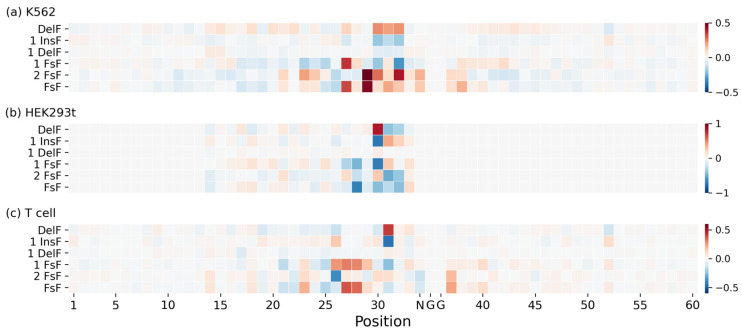
Visualization of the feature importance of nucleotide position in the target DNA sequence estimated by DeepIndel for six repair outcomes on the (**a**) K562, (**b**) HEK293t, and (**c**) T cell datasets. The nucleotide positions are arranged horizontally, whereas the datasets are arranged vertically. The color of each cell in the heatmap denotes the contribution of trinucleotides at each position for repair outcome prediction.

We next focused on analyzing the learned embedded representations using 1-mer and 3-mer for repair outcome prediction. Figure 5 shows the importance of the sequence content of nucleotides at each position learned by DeepIndel across these three datasets. Several interesting results can be observed: (i) We noted that the nucleotides at positions 22–33 are more important than those in other regions for predicting all editing results. Specifically, insertions and deletions mostly focused on positions 30–32, whereas frameshifts occurred mainly at positions 26–32, and especially at positions 27 and 29. These results imply that this region of the sequence plays critical roles in insertions and deletions. These position-dependent patterns coincide with the work of Jinek et al. [3] and Cong et al. [30], where their results illustrate that the 10–12 bp PAM-proximal seed sequence largely determines target accuracy. In addition, this finding is in agreement with a previous study indicating that the nucleotides 2 to 5 bp upstream of the PAM are critical for determining the editing precision of a target site [13,17]. (ii) Our model paid close attention to the expected cut site at position 30, which has a negative contribution to 1InsF. This observation is consistent with previous findings that the nucleotides 4 bp upstream of the PAM are the most influential in determining repair outcomes, at least for insertion [13,14,16]. Furthermore, we analyzed the positional effects of trinucleotides embedded using 3-mer (Supplementary Figure S5). Overall, the general patterns learned from trinucleotides agree with those of nucleotides. We noticed that trinucleotides at positions 26–33 are more important than other positions for all editing results. Besides, trinucleotides at positions 28–33 have positive contributions for DelF but negative contribution for 1InsF. Taken together, these results suggested that the nucleotides and trinucleotides surrounding the cut site are strong determinants of repair outcomes. This demonstrates the ability of our model to learn the positional preferences of target DNA sequences and generate interpretable visualizations of them.

## 3. Discussion

Accurate prediction of CRISPR/Cas9-mediated repair outcomes can enhance our understanding of the mechanisms of this system, thereby maximizing the potential and safety of CRISPR-based approaches. Although several computational predictions of repair outcomes have made significant progress recently, the performance still needs improvement. Here, we present an end-to-end multi-label regression model, called DeepIndel, for forecasting the outcomes of edits. To the best of our knowledge, this is the first application of BERT for the purpose of predicting repair results. We first split the target DNA sequence into overlapping *k*-mer vectors (*k* = 1, 2, 3) and mapped them into a 179-dimensional integer vector using the proposed token dictionary. Then, we trained the deep learning-based DeepIndel model to predict six Cas9-induced repair outcomes, including 1 InsF, 1 DelF, DelF, 1 FsF, 2 FsF, and FsF. Our ablation study showed that the proposed token dictionary is helpful for converting the DNA sequence into a numerical sequence. In addition, the BERT-based module in our model is critical for making predictions. Experiments on three public datasets showed that our model outperforms existing methods in terms of accuracy and robustness. Finally, we applied Deep SHAP to investigate the generalizable patterns of 1-mer, 2-mer, and 3-mer sequences at specific positions learned by our model for repair outcomes. Our visualization results illustrated that nucleotides and trinucleotides surrounding the cut site play a critical role in our model for predicting repair outcomes.

Our future work will focus on four aspects. First, we will expand the feature space. In the present study, we used target DNA sequence data only for model training and evaluation. Previous reports have shown that hand-crafted sequence features, such as nucleosome positioning, may influence Cas9 binding and cleavage [11,31]. A recent study also demonstrated that the outcomes of Cas9 editing depends on the chromatin state at the cut site [32]. Thus, incorporating other informative hand-crafted sequence features, such as nucleosome positioning and epigenetic factors, into our model may provide more insights regarding their potential for future improvements. Second, it has been reported that the distribution of repair outcomes differs across various cell lines [14]. Existing deep learning-based methods perform well on specific datasets but not on unseen ones. Therefore, there is still room for introducing more robust methods for datasets from various cell lines. Third, reasonable encoding schemes, which provide maximum biological characteristics information and reduce computational costs, will enhance the prediction interpretability and accuracy. Fourth, mounting evidence illustrates that Cas9-mediated repair outcomes are strongly dependent on the sequence context of the target site [9,13,15,33]. Our observations suggest that nucleotides and trinucleotides closer to the cut site are more important than dinucleotides for predicting repair outcomes. Inspired by this result, we plan to apply teacher–student-based knowledge distillation [34], which takes the sequence extracted by 1-mer and 3-mer as an input for the student model to investigate the contribution of nucleotides and their position-dependent preferences in each sequence, thus shedding more light on the interplay between target DNAs and their repair results. A deeper understanding of Cas9-induced mutations will enhance our ability to accurately orchestrate the positions and outcomes of genome editing. These are interesting topics that deserve to be explored in the future.

## 4. Materials and Methods

### 4.1. Data Resources

We used three datasets, including K562, HEK293t, and T cell, for model training and comparison. The K562 dataset was collected by Li et al. [22], which contains 35,129 target sequences along with corresponding labels. We collected the HEK293t and T cell datasets from public resources. After removing redundancy, the sizes of these two datasets were 4591 and 1603, respectively. Each entry in the datasets contains a 60 bp target DNA sequence along with six editing outcomes. Specifically, for each sequence in the dataset, we aligned the PAM sites at 33 bp so the cut site was at the center (30 bp) of all input sequences. If a sequence was shorter than 60 bp after PAM realignment, we applied the methods proposed by Liu et al. [25] to pad it into a 60 bp sequence. We computed the following six editing outcome statistics, namely the 1 bp insertion frequency (1 InsF), the 1 bp deletion frequency (1 DelF), the deletion frequency (DelF), the 1 bp frameshift frequency (1 FsF), the 2 bp frameshift frequency (2 FsF), and the total frameshift frequency (FsF). The detailed definitions of these six editing outcomes can be found in Supplementary Table S1.

### 4.2. Sequence Encoding

**Figure 6:**
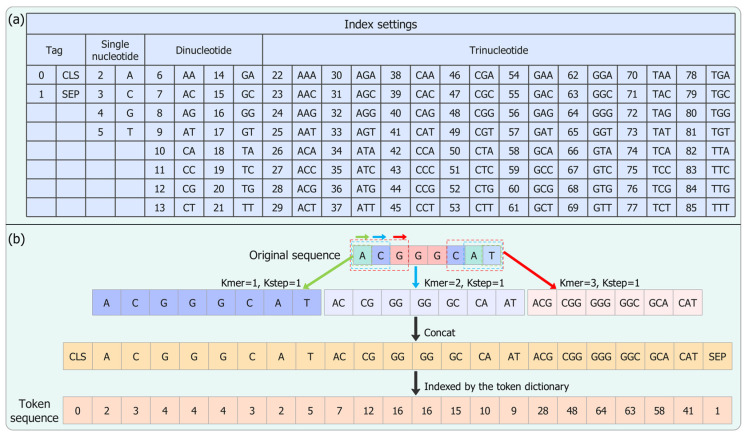
Sequence encoding for denoting the target DNA sequence. (**a**) Rules of setting index values for two special symbols (e.g., [CLS] and [SEP]), 4 single nucleotides, 16 dinucleotides, and 64 trinucleotides. (**b**) An example showing how to use the proposed token dictionary to encode the DNA sequence.

We split the input target DNA sequence of L_0 bp into overlapping *k*-mers using the sliding window approach before putting them into our model. We extracted all subsequences of length *k* with stride of 1, resulting in a *k*-mer sequence with length $L=\left(L_{0}-k\right)+1$, wherein all these k-mers were indexed by positive integers in set $C=\left[1,\;2,\;\cdots ,4^{k}\right]$. Specifically, each target DNA sequence was scanned using sliding windows with a k-mer length from 1 to 3 and a sliding step with a size of 1 to extract the nucleotides (*k* = 1), dinucleotides (*k* = 2), and trinucleotides (*k* = 3). Thus, the input sequence was separated into overlapping subsequences of length k. Notably, the semantic dictionary in BERT was designed for natural language text, which cannot be used directly to encode the sequence data. So, we constructed a token dictionary to convert each sequence into a numerical sequence as a suitable input for our model (Figure 6a). Two special tokens, including the classification token ([CLS]) and the separation token ([SEP]), were added to the front and end of the sequence, with values of 0 and 1, respectively. In total, 4 single nucleotides, 16 dinucleotides, and 64 trinucleotides were represented as unique tokens from 2 to 5, 6 to 21, and 22 to 85, respectively. The total size of the dictionary was 86 (${2+4}^{1}+4^{2}+4^{3}$). Therefore, we mapped the k-mer vectors in the obtained sequence into a numerical sequence with a length of 179 using the proposed token dictionary. The vectorization and concatenation of k-mers was computed in such way that their content was preserved.

Taking ‘ACGGGCAT’ in Figure 6b as an example, we first applied the *k*-mer (*k* = 1, 2, 3) with a sliding step of 1 to split it into the 1-mer (nucleotide), 2-mer (dinucleotide), and 3-mer (trinucleotide) sequences, including [A, C, G, G, G, C, A, T], [AC, CG, GG, GG, GC, CA, AT], and [ACG, CGG, GGG, GGC, GCA, CAT]. Then, we combined these three *k*-mer sequences into a vector with a length of 21 ([A, C, G, G, G, C, A, T, AC, CG, GG, GG, GC, CA, AT, ACG, CGG, GGG, GGC, GCA, CAT]). The two above-mentioned specialized tokens were added at the beginning and the end of the sequence. By applying the proposed token dictionary, this sequence was represented as [0, 2, 3, 4, 4, 4, 3, 2, 5, 7, 12, 16, 16, 15, 10, 9, 28, 48, 64, 63, 58, 41, 1].

### 4.3. DeepIndel Model

**Figure 7:**
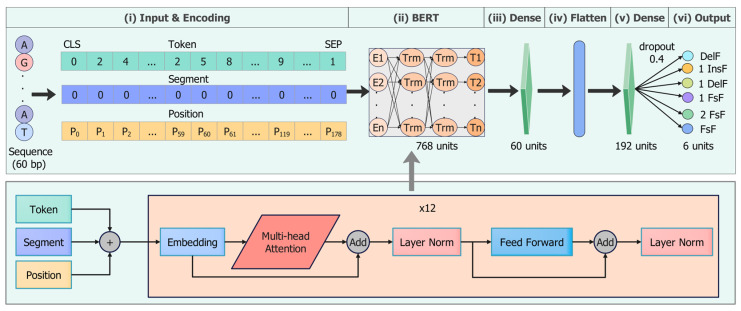
Illustration of DeepIndel and the transformer block in BERT. DeepIndel predicts the repair results by the following six stages. (i) A 60 bp DNA sequence centered at the cut site is used as the input to the model. The encoding layer converts the input sequence into a numerical sequence of length 179. (ii) The encoded sequence is fed into BERT to extract the hidden information. (iii) The outputs of BERT are input into the first dense layer for deeper feature extraction with the ReLU activation function. (iv) The outputs of the dense layer are flattened for further analysis. (v) The features are fed into the second dense layer to perform linear transformations. (vi) The output layer makes a multi-output regression prediction of repair outcomes.

We proposed a deep learning framework named DeepIndel for Cas9-induced repair outcome prediction. As depicted in Figure 7, the architecture of DeepIndel can be divided into seven parts, namely the (i) input, (ii) encoding, (iii) BERT, (iv) dense layer, (v) flatten layer, (vi) dense layers, and (vii) output layer. DeepIndel receives sequences of length 60 as inputs and produces the probabilities of six repair outcomes. The DNA sequence is encoded by embedding before being fed into the network. The BERT module is applied to extract the hidden information in sequence features, which consist of 12 transformer layers, 768 hidden units, and 12 attention heads with 110 million parameters. The encoded sequences learned from the BERT module become vectors with dimensions of 179 × 768, which are then fed into the dense layer with 60 neurons and a L1 regularization size of 1e-4. The outputs are flattened and fed into a dense layer with 192 neurons to perform linear transformation. These dense layers apply a ReLU non-linear activation function. The final output layer is a multi-output regression for each of the six editing outcome statistics. It contains 6 neurons, which are fully connected to the previous dense layer using the *sigmoid* activation function, for quantifying the propensity of six repair outcomes.

### 4.4. Model Training

We implemented the proposed method using python (3.8.18), keras (2.5.0), tensorflow-gpu (2.5.0), and transformers (4.30.2). The training and testing procedures were executed using a computer with Intel (R) Xeon (R) Silver 4316 CPU @ 2.30 GHz, Ubuntu 20.04 LTS, and one NVIDIA GeForce RTX 4090 with 24 GB of memory. Five-fold cross-validation tests were performed to compute the average performance, which contributes to the objective evaluation of the model. We optimized the binary cross-entropy loss function using an Adam optimizer and applied dropout for model regularization with a 0.4 dropout rate. We applied a learning rate of 1 × 10^−5^ to update the parameters during model training. The batch size and epoch were set to 64 and 200, respectively. Additionally, we implemented early stopping to terminate model training if the validation loss did not improve for 20 consecutive epochs.

### 4.5. Model Interpretability

In addition to accuracy and generalizability, we are also interested in the mechanism of our model. One of the main drawbacks of neural networks is their lack of interpretability. The multilayer non-linear hidden units of the BERT-based module in our model lead to a trade-off between complexity and interpretability. Several techniques have been developed to understand the black box nature of deep learning models. For instance, Deep SHAP [35] is a common visualization method that creates a background distribution in the input space. By applying this distribution to generate model predictions for a set of samples, the contributions that each feature makes to the output of the model can be calculated. A higher SHAP value means a higher contribution of the position to the result. For instance, Wang et al. [36] applied Deep SHAP to interpret the contribution of features of their model for sgRNA on-target activity prediction. To make the contribution of nucleotides comparable among these datasets, we rescaled the Deep SHAP values by standardization. The SHAP value for each position was the sum of the values for all samples at each position. The Deep SHAP values of different extracted features were the sums of important values at all 60 positions.

### 4.6. Performance Measurements

We used three quantitative indicators, including the Spearman correlation coefficient (SCC) [37], the Pearson correlation coefficient (PCC) [37], and Kendall’s rank correlation coefficient (KTau) [37], to evaluate the performance of DeepIndel. The SCC assesses the monotonic relationship between two variables, which is defined as follows:

(1) $$\begin{array}{c} \;SCC=1-\frac{6\sum_{i=1}^{n}{\left(x_{i}-y_{i}\right)}^{2}}{n\left(n^{2}-1\right)}\; \end{array}$$

where $x_{i}$ and $y_{i}$ are the *i*th elements in x and y, and n is the number of samples. The SCC ranges from −1 to 1, with −1 and 1 representing the strongest negative and positive correlation, respectively. The formula of PCC is as below:

(2) $$\begin{array}{c} \;PCC=\frac{\sum_{i=1}^{n}\left(x_{i}-\bar{x}\right)\left(y_{i}-\bar{y}\right)}{\sqrt{\sum_{i=1}^{n}{\left(x_{i}-\bar{x}\right)}^{2}}\sqrt{\sum_{i=1}^{n}{\left(y_{i}-\bar{y}\right)}^{2}}}\; \end{array}$$

where $\bar{x}$ is the mean of x and $\bar{y}$ represents the mean of y. KTau [37] evaluates the nonparametric correlation between two ordered variables and is given by the following formula:

(3) $$\begin{array}{c} \;KTau=\frac{\left(P-Q\right)}{\sqrt{\left(P+Q+T\right)*\left(P+Q+U\right)}}\; \end{array}$$

where P represents the number of positive correlated ranked pairs, Q denotes the number of negative correlated ranked pairs, T is the sum of all ranked pairs, and U is the number of remaining possible permutations. Since KTau does not rely on the specific distribution of the data, it is robust to outliers. In addition, we used the area under the receiver operating characteristic curve (AUC) [38] to quantify the performance, which was adopted in previous repair outcome prediction studies [22,25]. The value of the AUC is in [0, 1], where 1 is a successful performance.

## Figures and Tables

**Table 1:** Summary of DeepIndel model variants.

Model	Architecture
DeepIndel	Using the BERT-based module, two dense layers with 60 and 192 neurons, respectively.
DeepIndel-CNN	Replace the BERT module in our model with a CNN module.
DeepIndel-LSTM	Replace the BERT module in our model with a BiLSTM module.
DeepIndel-pre-trained	Replace the BERT module in our model with the pre-trained BERT-based module [27].

## Data Availability

Data is contained within the article.

## Supplementary Materials

The following supporting information can be downloaded at https://www.mdpi.com/article/10.3390/ijms252010928/s1.
