# Supplementary material for: DeepIndel: An Interpretable Deep Learning Approach for Predicting CRISPR/Cas9-Mediated Editing Outcomes
Source: Int J Mol Sci. 2024 Oct 11;25(20):10928. doi: 10.3390/ijms252010928 (PMC11507043; doi:10.3390/ijms252010928)
Supplement: Supplementary file 1 [file ijms-25-10928-s001.zip › ijms-3203147-supplementary.pdf]

International Journal of
Molecular Sciences

MDPI

Supplementary Table S1. Definition of six editing outcomes.

Editing outcome	Definition
Deletion frequency	$\frac{D}{D+I}$
1 bp insertion frequency	$\frac{I_{1bp}}{D+I}$
1 bp deletion frequency	$\frac{D_{1bp}}{D+I}$
1 bp frameshift frequency	The ratio of indel outcomes that induced a frameshift of 1 bp
2 bp frameshift frequency	The ratio of indel outcomes that induced a frameshift of 2 bp
Total frameshift frequency	The ratio of indel outcomes that induced a frameshift of 1 and 2 bp

Note: D, the number of deletions; I, the number of insertions; I_{1bp}, the number of 1 bp insertions; D_{1bp}, the number of 1 bp deletions; indels, insertions and deletions.

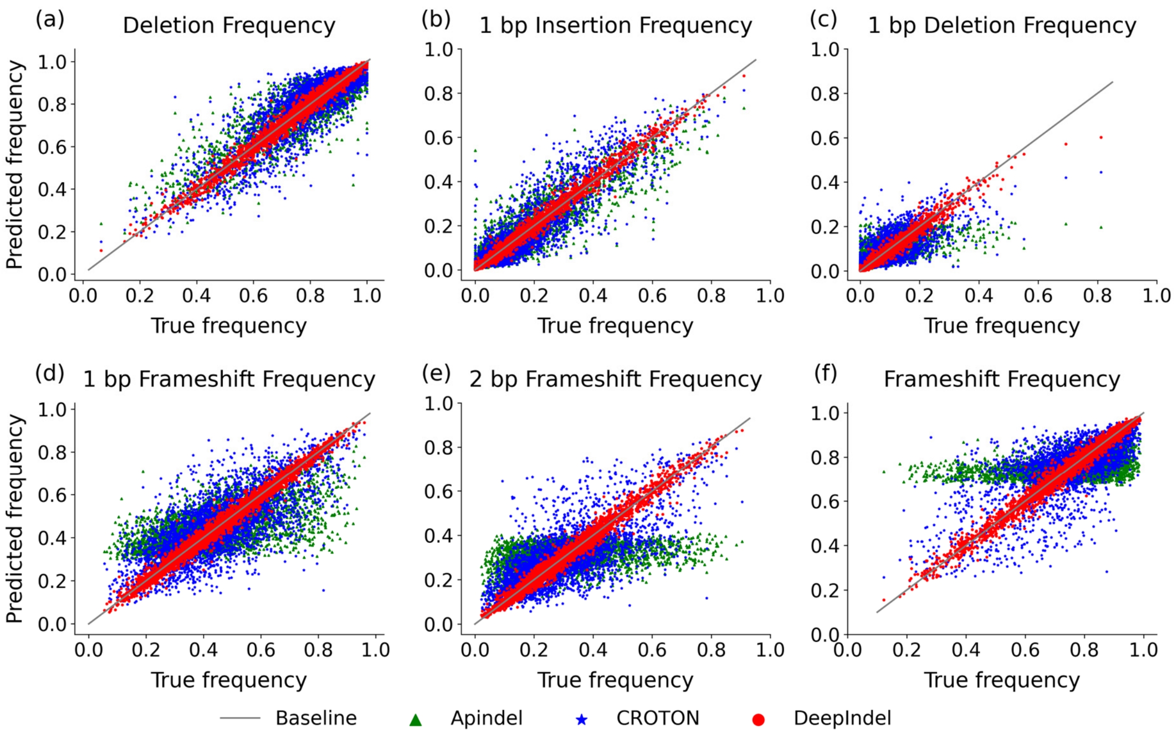

Supplementary Figure S1. The scatter plots show the correlations between true frequencies and predicted frequencies in terms of all prediction tasks on K562 test set of Deepindel and two deep learning-based methods. The x-axis represents true frequency, the y-axis indicates predicted frequency, and the baseline denotes ideal state where predicted frequency equal true frequency.

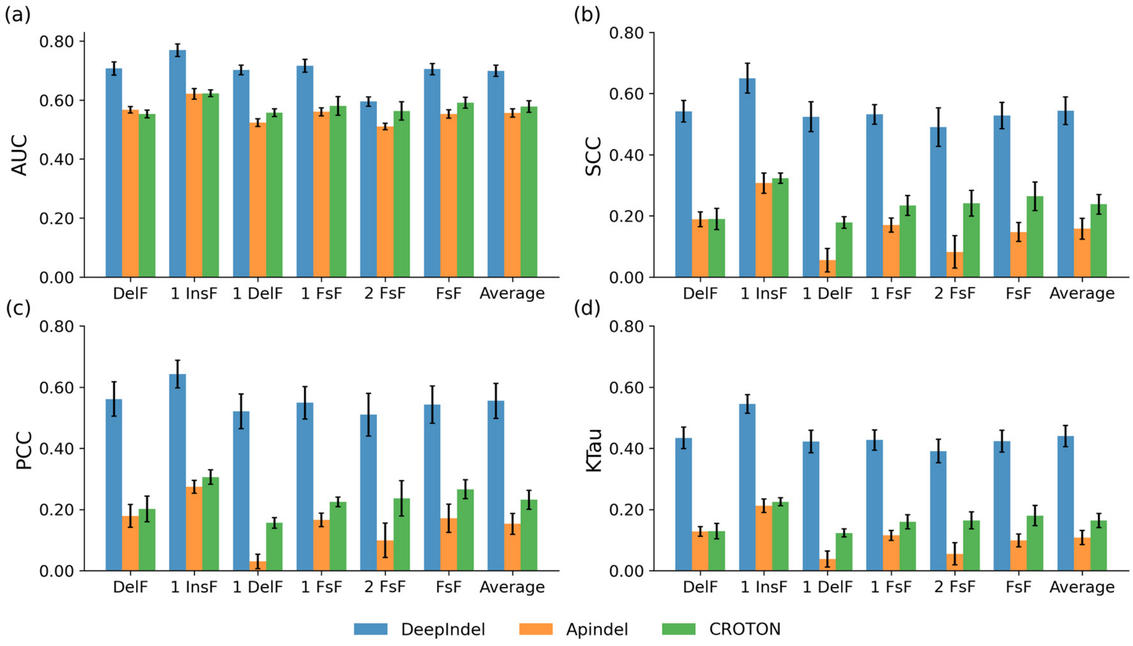

Supplementary Figure S2. The bar graphs show averaged AUC, SCC, PCC and KTau values of DeepIndel and two deep learning-based methods on HEK293t dataset under 5-fold cross-validation. The prediction methods are arranged vertically, whereas the repair outcomes are placed horizontally. Error bars represent the standard deviation.

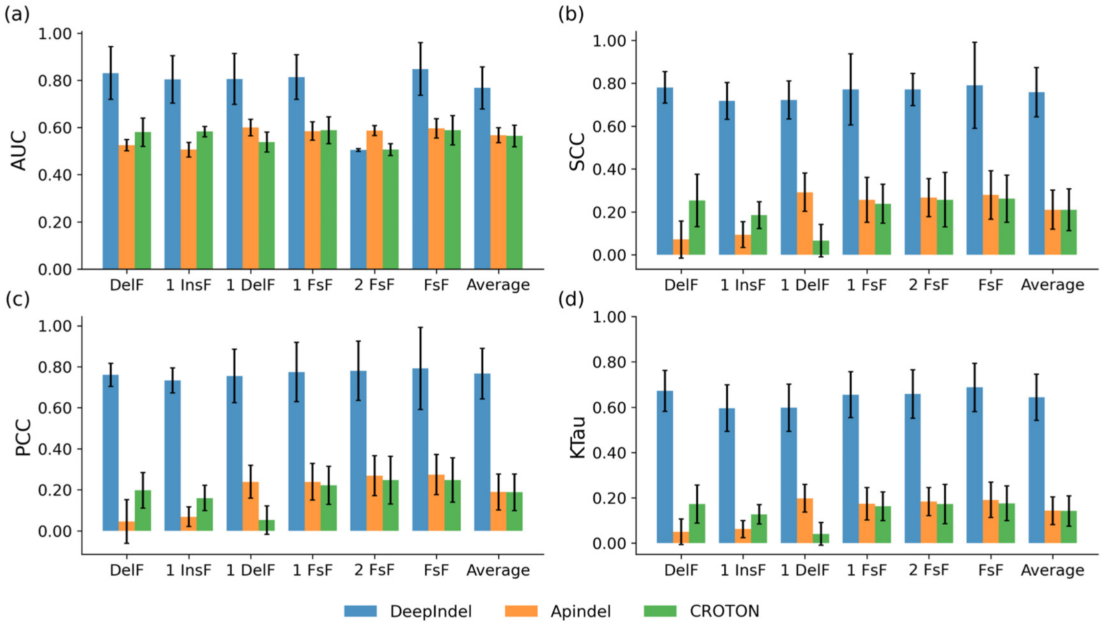

Supplementary Figure S3. The bar graphs show averaged AUC, SCC, PCC and KTau values of DeepIndel and two deep learning-based methods on T cell dataset under 5-fold cross-validation. The prediction methods are arranged vertically, whereas the repair outcomes are placed horizontally. Error bars represent the standard deviation.

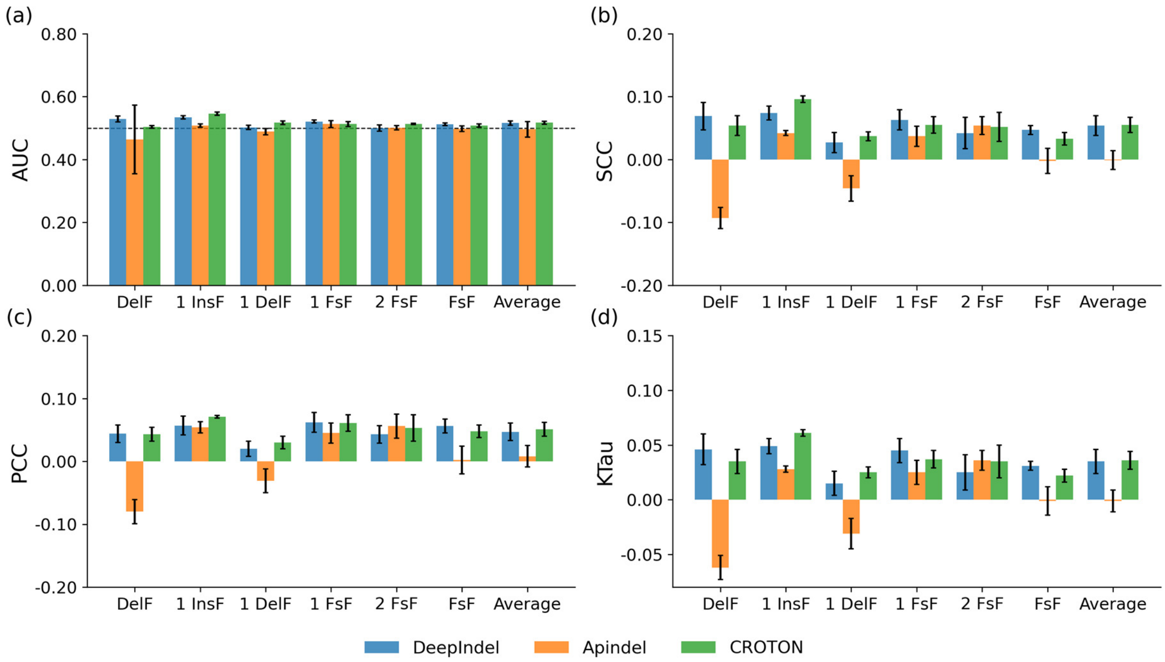

Supplementary Figure S4. The bar graphs show the (a) AUC, (b) SCC, (c) PCC and (d) KTau of DeepIndel with two existing deep learning-based CRISPR/Cas9 repair outcomes prediction methods (e.g., Apindel and CROTON) under cross-dataset validation. Models are trained on HEK293t dataset and tested their performance on T cell dataset. Error bars represent the standard deviation. The dotted line in (a) indicates an AUC of 0.5.

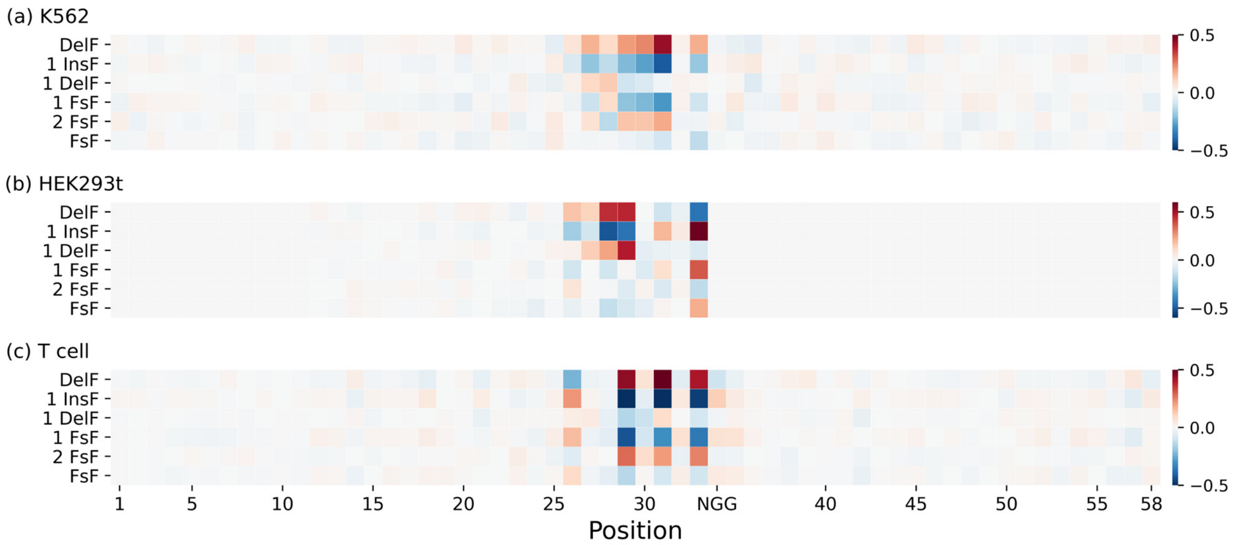

Supplementary Figure S5. Visualization of the importance of 3-mer for our DeepIndel trained on (a) K562, (b) HEK293t and (c) T cell datasets. The nucleotide positions are placed horizontally, whereas the datasets are arranged vertically. The colors denote the contribution of the nucleotide positions for repair outcomes prediction. The darker red indicates the positive contribution of the nucleotides at that position, whereas the darker blue means the more negative contribution.
